# A highly-selective layer-by-layer membrane modified with polyethylenimine and graphene oxide for vanadium redox flow battery

**DOI:** 10.1080/14686996.2023.2300697

**Published:** 2024-01-04

**Authors:** Saidatul Sophia Sha’rani, Mohamed Mahmoud Nasef, Nurfatehah Wahyuny Che Jusoh, Eleen Dayana Mohamed Isa, Roshafima Rasit Ali

**Affiliations:** a Department of Chemical and Environmental Engineering (ChEE), Malaysia–Japan International Institute of Technology, Universiti Teknologi Malaysia, Kuala Lumpur, Malaysia; b Advanced Materials Research Group, Center of Hydrogen Energy, Universiti Teknologi Malaysia, Kuala Lumpur, Malaysia

**Keywords:** Vanadium redox flow battery, layer by layer modification, polyethyleneimine, graphene oxide, perfluorosulfonic acid membranes

## Abstract

A selective composite membrane for vanadium redox flow battery (VRFB) was successfully prepared by layer-by-layer (LbL) technique using a perfluorosulfonic sulfonic acid or Nafion 117 (N117). The composite membrane referred as N117-(PEI/GO)*n*, was obtained by depositing alternating layers of positively charged polyethylenimine (PEI) and negatively charged graphene oxide (GO) as polyelectrolytes. The physicochemical properties and performance of the pristine and composite membranes were investigated. The membrane showed an enhancement in proton conductivity and simultaneously exhibited a notable 90% reduction in vanadium permeability. This, in turn, results in a well-balanced ratio of proton conductivity to vanadium permeability, leading to high selectivity. The highest selectivity of the LbL membranes was found to be 19.2 × 10^4^ S.min/cm^3^, which is 13 times higher than the N117 membrane (*n* = 0). This was translated into an improvement in the battery performance, with the *n* = 1 membrane showing a 4–6% improvement in coulombic efficiency and a 7–15% improvement in voltage efficiency at current densities ranging from 40 to 80 mA/cm^2^. Furthermore, the membrane displays stable operation over a long-term stability at around 88% at a current density of 40 mA/cm^2^, making it an attractive option for VRFB applications using the LbL technique. The use of PEI/GO bilayers maintains high proton conductivity and VE of the battery, opening up possibilities for further optimization and improvement of VRFBs.

## IMPACT STATEMENT

An innovative N117-(PEI/GO)n layer-by-layer membrane prepared for vanadium redox flow battery improved the balance between the proton conductivity and vanadium permeability, yielding a remarkable selectivity of 19.2 × 10^4^ S.min/cm^3^.

## Graphical abstract

**Figure:**
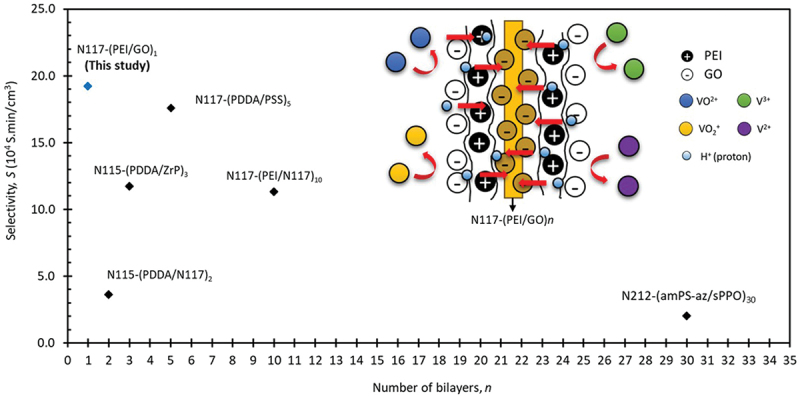

## 1. Introduction

The Nafion series, a perfluorosulfonic acid (PFSA) membrane, has been widely used in various applications including batteries like vanadium redox flow battery (VRFB), electrodialysis [1,2] and fuel cells [3]. This membrane is composed of a hydrophobic perfluorinated polyethylene backbone and hydrophilic sulfonic acid-terminated perfluorovinyl ether side groups. Its unique hydrophilic/hydrophobic nature gives it high proton conductivity and excellent stability in acidic and oxidizing vanadium electrolytes [4,5]. However, in VRFB, the vanadium permeability of such membranes leads to low coulombic efficiency (CE) and selectivity [6]. Therefore, improvements are needed to enhance the efficiency of the battery.

Various methods have been attempted to reduce the vanadium permeability of the PFSA membrane. One commonly used approach is to introduce additives or fillers to create a barrier for vanadium ions and improve ion selectivity [7]. These modifications can be achieved through sol–gel reactions [8], PFSA dissolution, or solution casting methods [9]. However, these methods have shown a decrease in membrane stability compared to the original PFSA membrane. Additionally, reproducing the modified membrane on a larger scale is challenging due to the multiple preparation steps involved [10]. As a promising solution to enhance ion selectivity, surface modification techniques like layer-by-layer (LbL) can be employed. LbL is a simple and straightforward method that involves applying alternating layers of oppositely charged polyelectrolytes through electrostatic interaction [11–13]. This technique has been widely used in various applications such as batteries, electrodes, supercapacitors, and sensors [14].

In the VRFB system, the development of LbL membranes primarily focuses on PFSA-based membranes, such as the Nafion series (e.g. N117, N115, and N212), hydrocarbon-based membranes such as sulfonated polyether ether ketone (SPFEK), and a combination of SPEEK with porous polytetrafluoroethylene (PTFE), and the most employed pair of polyelectrolytes were poly(diallyldimethylammonium chloride)/poly(sodium styrene sulfonate) (PDDA/PSS) [15–18]. These polyelectrolytes were not only used in VRFB applications but also found in other applications due to their wide availability, stability across a broad pH range, excellent solubility, and cost-effectiveness, which facilitates simpler modification procedures. Additionally, PDDA/N117 [19], PDDA/zirconium phosphate (ZrP) [20], chitosan/phosphotungstic acid (CS/PWA) [21] and azide containing quaternary ammonium polystyrene/sulfonated poly(2,6-dimethyl-1,4-phenylene oxide) (amPS-az/sPPO) [22] were also investigated for their compatibility as polyelectrolyte pairs for such membranes. In general, the selectivity of the prepared LbL membranes is influenced by the number of the incorporated bilayers. It was observed that as the number of bilayers increased, the reduction of vanadium ions occurred. This was attributed to the coverage of the ion-transfer pathway and the repulsion of vanadium ions due to the charges coming from the polycation. While this approach was successful in reducing the vanadium permeability of the membranes, a consistent observation emerged where the proton conductivity of the modified membrane decreased significantly as the number of bilayers increased, resulting in lower voltage efficiency (VE). The reduced proton conductivity was attributed to the repulsion forces generated by the bilayers, hindering proton movement through the membrane. The thickness of the membrane also affected proton conductivity and vanadium permeability, as a thicker membrane provided longer and more complex channels for ion transport [19]. Although the selectivity and CE of the battery improved, the proton conductivity and VE decreased, resulting in a lower overall efficiency (EE). To maintain high proton conductivity and VE, graphene oxide (GO) was suggested as a substitute for the commonly used polyanions, such as PSS and N117.

GO-modified membranes have attracted attention as a potential substitute material for VRFB applications. While GO-based materials have been utilized in various fields, their use as membrane materials, particularly for VRFB applications, is still relatively new. The utilization of GO as a membrane material in VRFB is appealing due to its high resistance to strong acidic solvents and exceptional mechanical stability [23,24]. Additionally, the presence of oxygenated functional groups (-O, -OH, and -COOH) makes GO highly hydrophilic, facilitating proton transportation by providing a hydrogen-bonded channel and enhancing membrane adaptability. Previous studies have evaluated the effectiveness of GO in impeding vanadium transport through IEMs [25,26]. These studies have shown that incorporating GO into the membrane can reduce vanadium permeability by creating a narrower channel for vanadium transport. Although GO has been extensively used as a filler for membrane modification, its role as a blocking agent on the membrane surface has received less attention [27]. While GO can decrease vanadium permeability, additional surface modifications are still necessary. In water, the carboxylic acid and phenolic hydroxyl groups on the 2D surface of GO become ionized, resulting in an inherent negative charge and functioning as a polyanion [28]. Consequently, GO alone cannot solely act as a blocking agent for vanadium ions, as its negative charge may induce vanadium ion permeation.

Polyethyleneimine (PEI), a commonly used polycation, is known for its effective blocking function on multivalent ions like vanadium [29] in VRFB and minerals in electrodialysis and desalination [30,31]. While many investigations have reported IEMs or porous membranes with different pairs of polyelectrolyte bilayers, to the best of our knowledge, none has reported on the LbL assembly of PEI/GO bilayers onto the surface of PFSA membranes for VRFB application. Like GO, PEI exhibits high stability at various pHs and has good solubility to allow for simpler modification procedure. The incorporation of PEI, rich in amine groups, was chosen to leverage on its advantageous properties as a polycation, which can reduce the vanadium permeation while at the same time improve the proton conductivity as previously seen in other work [32]. Both PEI and GO possess hydrophilic characteristics, making them favourable couples for making membranes with low vanadium permeation and high proton conductivity. This, in turn, is likely to yield exceptional selectivity, enhancing both CE and VE of the battery. The modified membrane properties were evaluated using analytical methods, and their performance was tested in VRFB test cells. Furthermore, the stability of the modified membranes under VRFB operation conditions was investigated.

## 2. Experimental

### 2.1. Materials

N117 membrane with 180 µm thickness was purchased from Fuel Cell Store series. Vanadium (IV) oxide sulfate hydrate, VOSO_4_.*n*H_2_O of 97% purity was purchased from Merck. Branched PEI (50 wt.% in H_2_O, Mw < 100,000) (Merck) was used as polycation, while GO (Graphenea Inc.) was used as polyanion. To prepare the GO polyanion solution, a 1.0 g of GO powder was dispersed in 100 mL deionized water (DI) and sonicated for at least 4 h.

### 2.2. Preparation of N117-(PEI/GO)n LbL membranes

The N117 membrane was used and pre-treated prior to LbL assembly following the standard protocol reported in literature [33]. This pre-treatment aims to protonate the surface of the membrane. The N117 membrane possesses a negatively charged surface due to the presence of numerous SO_3_^2-^ functional groups. Consequently, the pristine membrane could be effectively modified by applying a positively charged PEI layer, followed by the deposition of a negatively charged GO layer. The successful deposition of both the PEI and GO monolayers on the N117 membrane is referred to as PEI/GO bilayers, *n*.

Following the pre-treatment procedure, the membrane surface was self-assembled with PEI/GO bilayers by sequentially dipping the membrane into 1.0 wt% of PEI solution and 1.0 wt % of GO solution. The polyelectrolyte solutions were used without any pH adjustment. The dipping time in polyelectrolytes was maintained for 10 min at room temperature. The membranes were washed five times to remove all the excess polyelectrolytes after each dipping step. The process was repeated to obtain the desired number of bilayers. The pristine N117 (*n* = 0) was used as a reference and counterpart membranes modified with PEI/GO bilayers with *n* = 1, 3, 5, or 7 were fabricated.

### 2.3. FTIR, FESEM, and XRD analyses of membranes

The Fourier transform infrared (FTIR) absorption spectra of the pristine and LbL membranes were recorded on a Spectrum 100 FT-IR (Perkin Elmer, USA) spectrophotometer with a frequency range of 4000–400 cm^−1^ with 32 scans and 4 cm^−1^ resolutions. Images of the surface and cross-section of the membranes (coated with gold) were recorded with an accelerated voltage of 5.0 kV and 100–10,000× magnification using a field emission scanning electron microscope (FESEM, GEMINI500, Germany). The samples were fractured after dipping in liquid N_2_ to investigate the cross-section. The membrane thickness was measured through cross-section FESEM images using ImageJ. The structural changes of the membranes were investigated by X-ray diffraction (XRD) analysis using a PANalytical Xpert Pro (UK) Xpert Pro analyser (λ = 0.15406 nm) in the range of 2θ = 5°– 80° with 2°/min at 45 kV and 20 mA.

### 2.4. Water uptake (WU) and swelling ratio (SR)

Water uptake and swelling ratio of the membranes were measured by recoding the changes in weight and dimension before and after immersion in de-ionized (DI) water for 24 h at 30°C according to Equations (1) and (2).

(1) $$WU=\frac{W_{wet}-\;W_{dry}}{W_{dry}}\;\times \;100\%$$

(2) $$SR=\frac{X_{wet}-\;X_{dry}}{X_{dry}}\;\times \;100\%$$

where, *W*_*wet*_ is the weight of swollen membrane, *W*_*dry*_ is the weight of dry membrane, whereas X_*wet*_ and X_*dry*_ are the thicknesses or lengths of swollen and dry membrane, respectively.

### 2.5. Ion exchange capacity (IEC)

To measure the IEC, the membranes were first immersed in 1.0 M sodium chloride (NaCl) for 24 h followed by titration of the released chloride ions with 0.01 M sodium hydroxide (NaOH) using phenolphthalein as an indicator. The IEC value is calculated using Equation (3).

(3) $$IEC=\frac{\Delta V_{NaOH}C_{NaOH}}{W_{dry}}$$

where $\Delta V_{NaOH}$ and $C_{NaOH}$ are the volume (ml) and concentration of NaOH solution (M), respectively.

### 2.6. Proton conductivity, vanadium ion (VO^2+^) permeability, and selectivity

The through-plane proton conductivity of the membranes was also measured using electrochemical impedance spectroscopy (EIS) (MTS740, Scribner Assoc., USA). For this test, the membrane was placed between two platinum electrodes attached with carbon paper (Sigracet 29 BC) and measured with PSM1735 FRA (Newton) electrochemical interface from 1 Hz to 1 MHz at 30°C and 100% relative humidity. Prior to the measurement, the membranes were immersed with 2.0 M H_2_SO_4_ at room temperature (28 ± 3°C) for 48 h. The proton conductivity of the membranes was estimated from the resistance (*R*) obtained from the Nyquist plot, which was obtained by ZPlot software. The proton conductivity (σ) is calculated using Equation (4):

(4) $$\sigma \left(\frac{S}{cm}\right)=\frac{L\left(cm\right)}{R\left(\Omega \right).A\left(cm^{2}\right)}$$

where *L* is the distance between the two electrodes of the cell and AR denotes the area resistance.

The vanadium ion permeability was measured using vanadium (IV) in a custom-made diffusion cell with two chambers of 50 ml capacity. The membrane active area was 2.0 cm^2^. The left and right sides of the diffusion cell were filled with 1.5 M VOSO_4_ in 3.0 M H_2_SO_4_ and 1.5 M Na_2_SO_4_ in 3.0 M H_2_SO_4_, respectively. Both solutions were magnetically stirred at 220 rpm to avoid concentration polarization during the test. The solution samples were collected at pre-determined time intervals, and the concentration of VO^2+^ was measured using the UV-Visible spectrometer (UV − 1800 Shimadzu) with the help of a prepared standard curve of known VO^2+^ concentration. In particular, the concentration of resultant VO^2+^ ions was calculated from the slope of VO^2+^ ion concentration versus diffusion time, *t* plot and represented by Eq. 5.

(5) $$C_{R}=\frac{AP}{V_{R}T}C_{0}\left(t-t_{0}\right)$$

where *C*_*0*_ is the total concentration of VO^2+^ ions at the initial time *t*_*0*_ and *V*_*R*_ is the volume of solutions. *T* and *A* are the membrane thickness and effective surface area, respectively. The selectivity was obtained by calculating the ratio of proton conductivity to permeability of vanadium (σ_H_^+^_/_*P*_VO_^2+^).

### 2.7. VRFB single cell performance with present membranes

To conduct VRFB testing, the vanadium electrolyte was prepared using vanadium (IV) oxide sulfate hydrate. A solution of 1.5 M V^4+^ in 3.0 M H_2_SO_4_ was prepared and utilized as the initial electrolyte for both the positive and negative compartments. Each compartment had a volume of 30 ml, and these solutions are cyclically pumped through the cell at a flow rate of 5 mL/min. The charge-discharge test of the cell was carried out at current densities of 40, 60, and 80 mA/cm^2^. The charging voltage was limited to 1.65 V, while the discharging voltage was limited to 0.8 V. The cycling process was recorded using a battery analyzer (BTS-8, MTI Corp., Korea). The coulombic efficiency (CE), voltage efficiency (VE), and energy efficiency (EE) were calculated as follows:

(6) $$CE=\frac{\int I_{d}dt}{\int I_{c}dt}\;\times \;100$$

(7) $$VE=\frac{\int V_{d}dt}{\int V_{c}dt}\;\times \;100$$

(8) $$VE=\frac{EE}{CE}\;\times \;100$$

The self-discharge test was also conducted to investigate and support the finding from the permeability testing. The test was performed by charging the cell at 40 mA/cm^2^ and ended when the open-circuit voltage (OCV) dropped to 0.8 V. In addition, the stability test was also carried out by running at least 100 charge-discharge cycles.

## 3. Results and discussion

### 3.1. Changes in chemical structure of membrane

**Figure 1.**
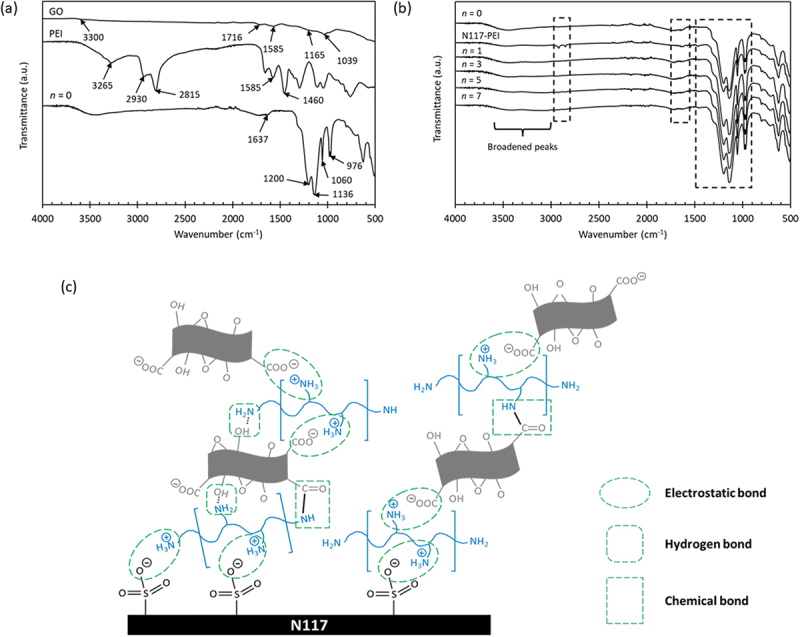
FTIR spectra of (a) N117, PEI and GO and the (b) LbL membranes and (c) the possible type of interactions of PEI/GO on N117 surface.

The FTIR spectra of the pristine N117 (*n = 0*) membrane and both polyelectrolytes, which were used as references, are depicted in Figure 1(a) whereas those of LbL membranes with different bilayers are shown in Figure 1(b). The spectrum of N117 (*n = 0*) shows a broad peak in the 3000–3600 cm^−1^ range, indicating the presence of O-H stretching vibration involved in H-bonding with moisture absorbed by the membrane. Additionally, a peak at 1630 cm^−1^ corresponds to the O-H bending vibration of free water molecules can be observed. The fluorinated backbone of N117 membrane exhibits symmetric and asymmetric C-F bond stretching vibrations at 1136 and 1200 cm^−1^, respectively. The presence of S-O groups of sulfonic acid was observed at 1060 cm^−1^, and C-O-C stretching was shown at 976 cm^−1^ [34]. In the case of PEI, characteristic features of branched C-H are observed at 1460, 2815, and 2930 cm^−1^. Furthermore, N-H bending vibration and N-H stretching vibration can be seen at 1585 cm^−1^ and 3265 cm^−1^, respectively [32,35,36]. The GO spectrum shows a broad band of strong OH stretching mode at around 3300 cm^−1^. An absorption peak of C=C stretching is also observed at 1585 cm^−1^. Stretching vibration modes of C-O, C-OH, and C=O can be seen at 1039, 1165, and 1716 cm^−1^, respectively [32,37].

Upon membrane modification with the PEI monolayer, additional peaks in the range of 2800–2930 cm^−1^, corresponding to the C-H from the branched PEI, are observed as depicted in Figure 1(b). However, when the GO monolayer is added, the peak appears to diminish, causing other peaks around 1580–1720 cm^−1^ to slightly deviate and increase in intensity. These changes are likely due to the presence of C=O and C=C originated from the GO, which covers the surface of the N117-PEI membrane. Moreover, the intensity of the peaks in the range of 900–1700 cm^−1^ increased when the GO monolayer was formed. Broadened peaks were also observed in this region of 3000–3600 cm^−1^ when GO was added, indicating an increased water absorption by the highly hydrophilic GO. Figure 1(c) illustrates the possible types of interactions that most likely took place during the GO and PEI LbL deposition. Previous studies have reported that the interconnection between PEI and GO nanosheets primarily occurs through electrostatic interactions, which act as the main driving force for the formation of PEI/GO bilayers. Other intermolecular bonds, such as hydrogen bonds and chemical bonding, may also be involved [38,39].

### 3.2. Changes in morphology of membranes

**Figure 2.**
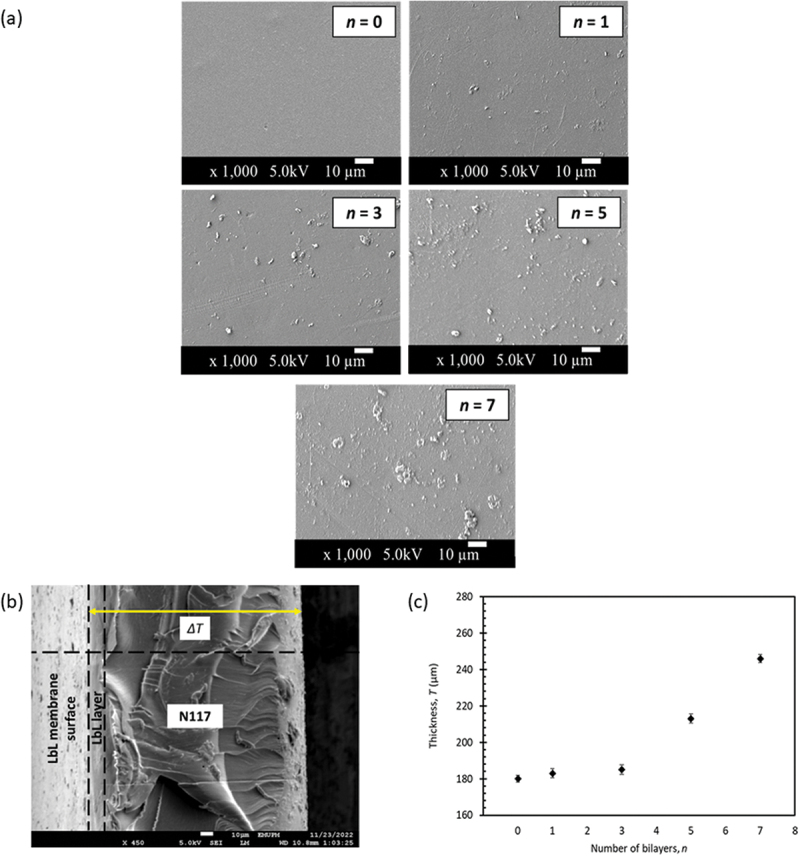
FESEM images (magnification x1000): (a) surface morphology of the LbL membranes, (b) cross-sectional thickness and (c) changes in thickness with number of bilayers.

The surface morphology of the N117 membranes with different bilayers was examined using FESEM, and the obtained images are presented in Figure 2(a). N117 pristine (*n = 0*) membrane was used as a control. The pristine membrane appeared smoother compared to other LbL membranes that exhibited denser and rougher structures with small agglomerations. These agglomerations increased with the increase in the amount of PEI/GO that gradually increased depositing more *n* layers. The rough surface observed on the LbL membranes can be attributed to the presence of incomplete unfolded GO nanosheets as reported elsewhere [40]. This indicates that the deposition of PEI/GO bilayers became less uniform when a large amount of GO is deposited.

Determining the thickness of each PEI/GO bilayer in LbL membranes and identifying them in the FESEM cross-section is challenging. This difficulty arises from the unclear appearance of the layers in the FESEM image, as the growth of the PEI/GO bilayers does not exhibit a distinct layer-to-layer overlay. Therefore, the cross-section image was primarily used to estimate the overall thickness of the LbL membranes rather than precisely measure the thickness of individual PEI/GO bilayer. Although measuring each bilayer is challenging, the growth of the LbL film becomes apparent with an increase in *n*, as shown in Figure 2(b,c). The membrane thickness appears to exhibit an exponential growth with the variation of *n* layers indicating that the growth of the LbL film under the given deposition conditions is non-linear and this might be caused by the variation of the types of formed bonds.

**Figure 3.**
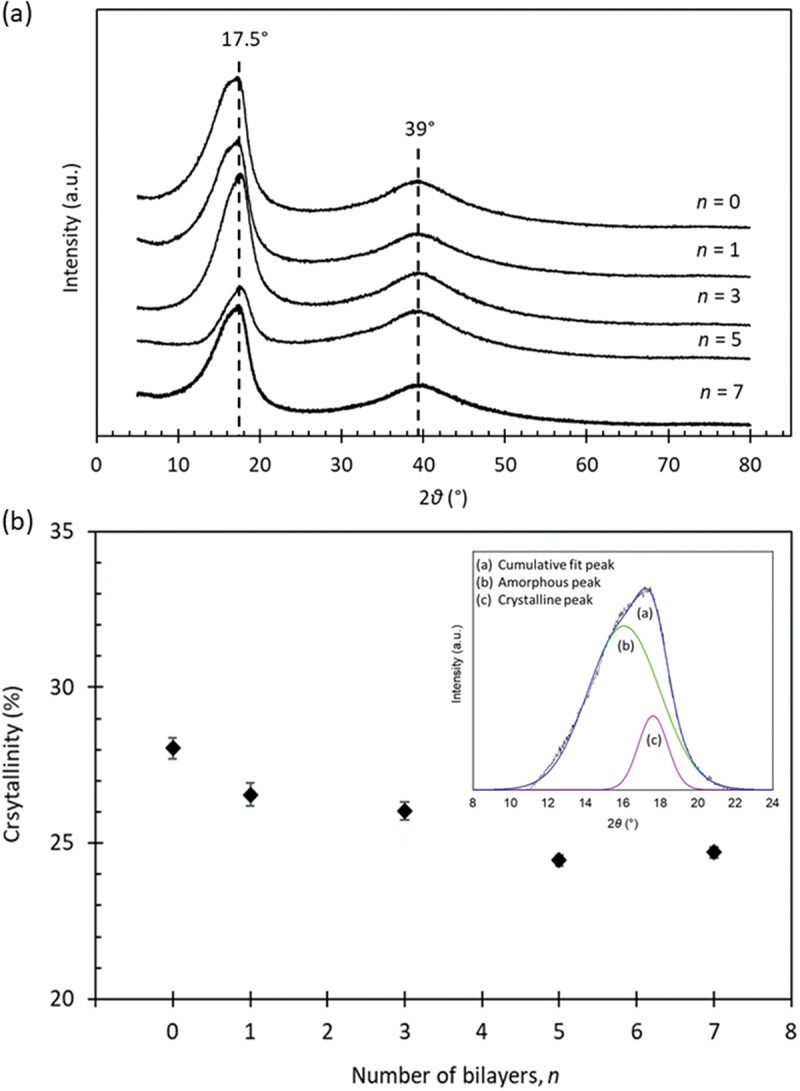
(a) XRD and (b) crystallinity of the LbL membranes. The inset in (b) shows the XRD of n = 1 containing cumulative fit, amorphous and crystalline peaks for determining the crystallinity (%) of the membrane.

Figure 3(a,b) and illustrate the XRD diffractograms of LbL membranes with different *n* layers and variation of the crystallinity of LbL membranes with number of layers. The *n* = 0 membrane exhibited a noticeable peak at 2θ = 17.5° that corresponds to a PFSA crystalline peak coupled with a halo at 2θ = 39° both of which confirmed the semi-crystalline nature of the perfluorocarbon backbone N117 membrane [34,41]. As the number of layers increased, the intensity of the crystallinity peaks became slightly smaller with a minor shift and such a trend is due to the transition towards a lower crystalline by dilution with the incoming amorphous bilayers [42]. It is noteworthy mentioning that no extra peaks were observed at 2θ = 12° in the modified membrane, suggesting that the layered structure of GO was not maintained during the LbL deposition process. These results provide an evidence for a successful consecutive alternative deposition of PEI and GO, indicating a favorable interaction between the membrane and the PEI/GO bilayers [43].

The reducing crystallinity trend of the LbL membranes was further evaluated by analyzing the relative proportions of the crystalline and amorphous phases based on the diffraction peak occurring at 2θ = 12–20°. The broad peak within this range was examined to identify two distinct peaks originating from X-ray scattering: one at 2*θ* = 16° associated with the hydrophilic region, and another at 2*θ* = 17.5° linked to the hydrophobic perfluorocarbon backbone chain of the N117 structure [44]. The LbL membranes exhibited a reduction crystallinity trend with the increase of *n*. For instance, a 3.3% decrease was observed for the *n* = 7 membrane. This reduction in crystallinity is believed to be caused by the interspersion of surrounding polyelectrolyte chains between the interlayers of GO [45].

### 3.3. Water uptake, swelling ratio, and ion exchange capacity

Ion-exchange capacity (IEC), water uptake (WU), and swelling ratio (SR) are closely associated with the exchangeable ions present in the polymeric membrane. These factors exert a substantial influence on the overall performance of the membranes within the VRFB system. Moreover, water absorption plays a pivotal role in proton conduction, but an excessive amount of absorbed water can result in excessive swelling, thereby reducing selectivity and compromising the mechanical properties of the membrane. Table 1 demonstrates the impact of the number of PEI/GO bilayers on the WU, SR, and IEC of the LbL membranes. The results indicate that the LbL membranes exhibit a slight enhancement in IEC and a noticeable 17% increase in WU compared to the *n* = 0 membrane. Such an increase in WU can be attributed to the compatible pair of the polyelectrolytes in a way leading to increased hydrophilicity in the LbL membranes. As previously seen from the FTIR analysis, there is evidence in the LbL membrane in which the membrane becomes more hydrophilic. Both PEI and GO possess functional groups (-OH and -NH_2_) with water affinity, enabling strong interaction through hydrogen bonding. Therefore, the chemical structure of these material readily absorbs and holds water even with small amounts of PEI/GO bilayers. Although PEI/GO bilayers contribute to increased WU, they appear to restrict the swelling of the LbL membranes. This observation is attributed to the constrained migration and rearrangement of the polyelectrolyte chain caused by the neighbouring GO nanosheet layer’s confinement effect [45]. The observed increase in WU is reasonable, considering that excessive water uptake can lead to significant permeability issues and render the membrane dimensionally unstable [46].

**Table 1.** IEC, water uptake, and swelling ratio of LbL membranes.

*n*	IEC (meq/g)	Water Uptake (%)	Swelling Ratios (%)	
			In-plane *S*_*ǁ*_	Through-plane *S*_*⊥*_
0	0.88	14.8	10.0	11.1
1	0.89	18.2	10.9	9.0
3	0.91	18.2	6.0	8.9
5	0.91	19.0	9.7	8.2
7	0.91	18.2	7.8	6.4

Several previous studies have shown that the LbL membranes used in VRFB and fuel cells generally exhibit a decrease in WU and IEC values [18,47,48]. This decrease is attributed to the limited availability of functional groups that can facilitate ion-exchange reactions and form the LbL chain structure through electrostatic interactions. Most of these studies used PSS as the polyanion, which resulted in relatively lower hydrophilicity compared to when GO was used. While the sulfonic acid groups in PSS contribute to its hydrophilicity to some extent, they are not as effective as the oxygen-containing groups in GO in forming hydrogen bonds with water. Additionally, the use of other polyanions such as PDDA/CNC and PDDA/ZrP in LbL membranes was found to decrease hydrophilicity and increase hydrophobicity [20,49].

Interestingly, when GO was used as a modifier in the casting solution technique for membrane making, the IEC and WU values of the composite membrane generally increased with an increase in SR. During the casting process, GO nanosheets are randomly dispersed within the polymer matrix, creating a network of interfacial interactions. This allows more water to pass through the membrane, resulting in a higher swelling ratio [25,50,51]. Researchers have attempted various approaches to address the issue of high SR when GO is used. One effective method is the application of the LbL modification technique, which not only reduces the SR but also improves the IEC and WU. By using a polycation, the interlayer interaction between GO is enhanced through electrostatic interactions, which restricts the mobility of the polymer chains. As a result, the SR of the LbL membrane decreased when GO is deposited as the polyanion [52].

### 3.4. Proton conductivity, vanadium ion permeability, and selectivity

**Figure 4.**
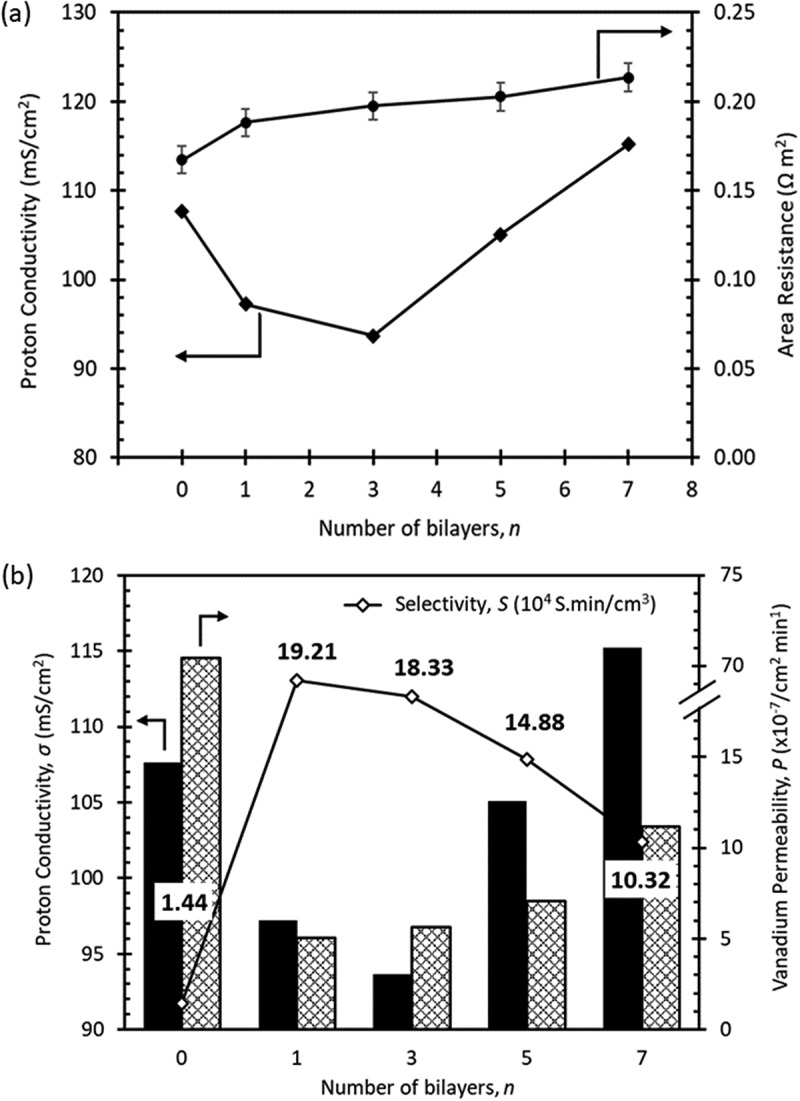
Effect of number of deposited layers on (a) proton conductivity and area resistance and (b) selectivity in terms of proton conductivity and vanadium permeability of LbL membranes.

The performance of a battery is determined by various factors, including AR, which affects both charge transfer and mass transfer rates of the membrane. In the case of VRFB, ions move across the membrane, making the through-plane conductivity more relevant for measurement. Theoretically, membranes with the same ionomer chemical structure exhibit an increase in AR with thickness [53]. Figure 4(a) shows the relationship between AR and proton conductivity in LbL membranes. A gradual increase in AR with the thickness of the membrane increases can be obviously observed. Surprisingly, the proton conductivity of the LbL membrane did not follow the same trend as AR. This discrepancy suggests that either AR or the increase in membrane thickness influences proton conductivity. However, when the membrane reached a limit of *n* = 3, the subsequent number of bilayers showed an increase in proton conductivity despite having slightly higher AR. This indicates that using GO in the LbL membrane has a more significant impact on enhancing proton conductivity. As mentioned earlier, GO improves the hydrophilicity of the LbL membrane, enhances WU, and facilitates the transportation of more protons through the membrane [37,45,54,55]. With the PEI/GO bilayers, proton conduction most likely occurs by proton hopping known as the Grotthuss mechanism in the network formed between the amine group of PEI and GO. The proton transfer is also facilitated by the network of hydrogen bonds formed by the amine groups of PEI interactions and the solvation of SO_3_^−^ present in N117.

The comparison between the vanadium permeability of the *n* = 0 membrane and the LbL membranes revealed a significant decrease (by 90%) in the permeation of VO^2+^ ions after modification is depicted in Figure 4(b). This indicates that the incorporation of PEI/GO bilayers on the membrane surface restricts the passage of VO^2+^ ions by introducing additional repulsive forces from both PEI and GO charges [36]. Furthermore, it has been established that thicker membranes generally exhibit lower vanadium permeability due to the longer pathway required for the ions to traverse through the membrane [56]. The combination of these repulsive forces, along with the presence of longer and convoluted channels in the LbL membranes, ultimately leads to a reduction in vanadium permeability.

Selectivity, denoted as *S* which equals to the measurement of the ratio between proton conductivity and vanadium permeability (σ_H+_/P_VO2+_). In VRFBs, high selectivity is indicative of superior membrane performance. However, striking a balance between proton conductivity and permeability presents a challenge, as both factors greatly impact battery performance. There is a conflict between strong selectivity and good proton conductivity, as an increase in one typically leads to a decrease in the other. Among the LbL membranes, the membrane with *n* = 1 demonstrated the highest selectivity (19.2 × 10^4^ S.min/cm^3^), with selectivity gradually decreasing as *n* increases. The membrane with *n* = 0 exhibited the lowest selectivity value of 1.44 × 10^4^ S.min/cm^3^. Since the selectivity of the membranes decreases significantly with *n*, further testing was conducted on the membrane with the highest selectivity (*n* = 1), the middle ones (*n* = 5), and the membrane with *n* = 0 for comparison.

**Figure 5.**
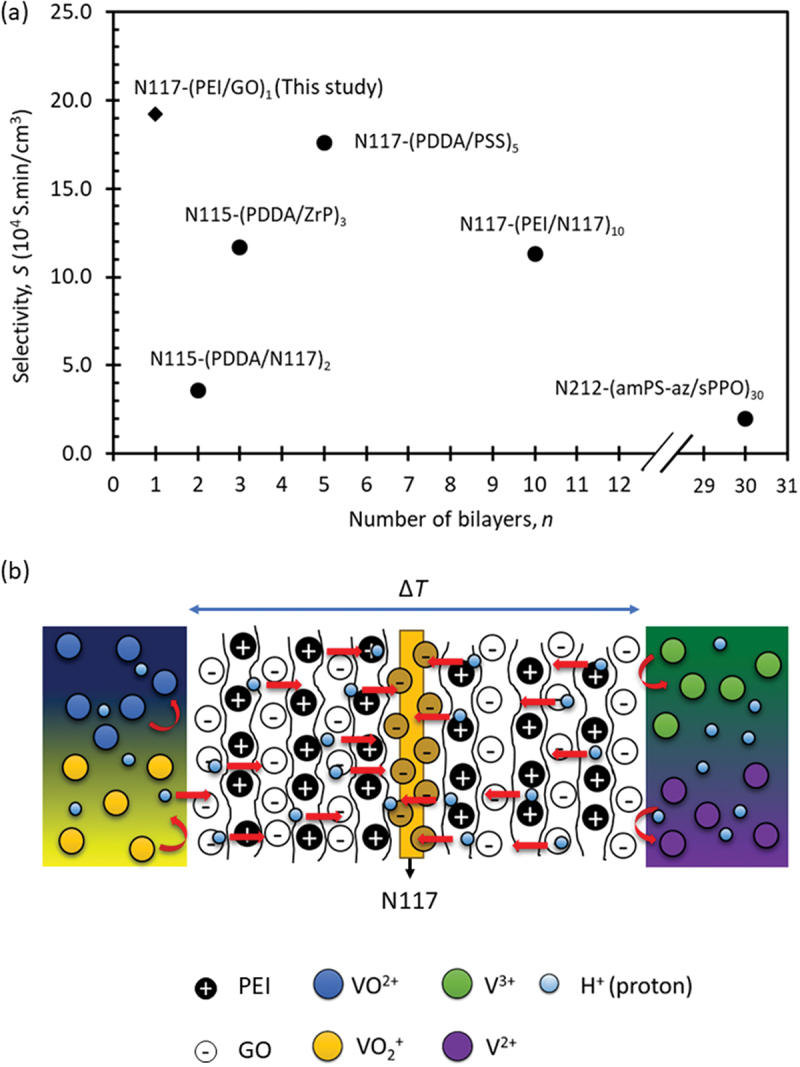
(a) Selectivity of present Nafion based LbL membranes compared to counterparts in previous studies and (b) plausible transport mechanisms for both proton and vanadium ions through present N117-(PEI/GO)n membrane.

The selectivity values of the present LbL membranes were compared to previously report counterparts in membranes modified with different bilayer polyanions. Figure 5 shows the variation of selectivity with number of layers for Nafion-based LbL membranes with different polyanion pairs reported in literature [15,19,20,22,29]. It can be observed that the membrane prepared in the present study, N117-(PEI/GO)_1_, had the highest selectivity value followed by N117-(PDDA/PSS)_5_ and N115-(PEI/N117)_10_ membranes. However, the PEI/GO polyanions pair achieved maximum selectivity with just one bilayer. In contrast, previous studies using PFSA-based membrane and GO as a filler modified with casting technique, specifically GO@PFSA-PTFE, demonstrated approximately four times lower selectivity compared to the value obtained in this study, which was approximately 4.7 × 10^4^ S.min/cm^3^ [53].

Figure 5(b) depicts the possible mechanism for proton and vanadium ions within the present membrane. The proton conduction through the LbL membranes occurs via proton hopping or Grotthus mechanism between the amine group of PEI and GO. The amine groups of PEI interact via hydrogen bonds or protonation with the SO_3_^−^ from N117 as previously described [29]. Then, the water molecules in the electrolyte can be effectively attracted by the epoxide and hydroxyl functional groups onto the surface of the GO sheets to form a hydrogen-bonding network. As a result, the proton can hop freely between both adjacent and non-adjacent hydroxyl functional groups through these hydrogen bonding networks [57]. However, the PEI/GO layers on N117 can strongly block vanadium ions from passing through in two main ways: they cover the polar clusters on the Nafion surface and create a repelling force due to the positive charge of the PEI layer.

### 3.5. VRFB performance

**Figure 6.**
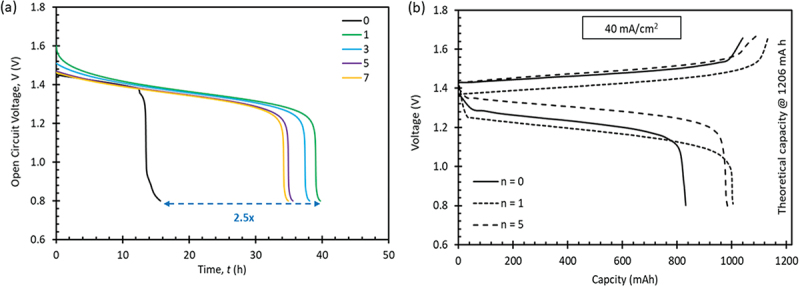
(a) OCV curve of the LbL membranes and (b) charge-discharge capacity profile of the selected membranes.

Figure 6(a). A comparison between the *n* = 0 membrane and the LbL membranes revealed that the latter exhibited significantly longer time spans for the voltage to decrease to 0.8 V. Additionally, the voltage of the LbL membranes decreased notably to 1.2 V before sharply dropping to 0.8 V. This suggests that the presence of the PEI/GO bilayers was highly effective in preventing excessive vanadium permeability during actual VRFB operation. The OCV results aligned with the vanadium permeability trend, confirming that the *n* = 1 membrane had the lowest vanadium permeability among the LbL membranes.

Figure 6(b) depicts the standard charge-discharge patterns of the chosen membranes under a current density of 40 mA/cm^2^. Among the three tested membranes, the membrane with *n* = 1 demonstrated the highest capacity, approximately four times greater than the membrane with *n* = 0. Importantly, the discharge capacity curve for the *n* = 1 membrane closely matched the size of the charge capacity curve, indicating a higher Coulombic Efficiency (CE) for this membrane. A similar trend was observed for the *n* = 5 membrane. Conversely, the membrane with *n* = 0 exhibited a significantly smaller discharge capacity curve compared to the charge capacity curve, suggesting severe vanadium permeability issues. These charge-discharge capacity curve profiles accurately reflect the vanadium permeability and OCV trends observed in this study, aligning with the findings from the physicochemical analysis. Consequently, the membrane with *n* = 1 is the optimal choice for preventing vanadium permeability, potentially leading to improved performance and increased lifespan.

**Figure 7.**
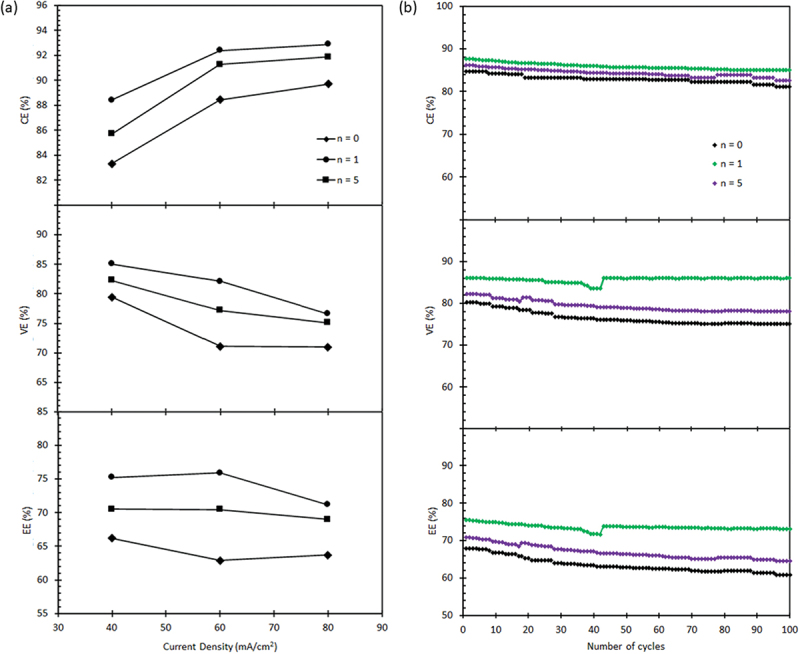
(a) The charge-discharge current density on CE, VE and EE of the VRFB with the (b) 100 cycles of the selected membranes.

Figure 7(a) illustrates the CE, voltage efficiency (VE), and energy efficiency (EE) of the chosen membranes in the VRFB single test at various charge-discharge current densities. All the tested membranes exhibited a permeability trend consistent with the findings of this study. Specifically, the membrane with the highest vanadium permeability showed the lowest CE, and vice versa. At a current density of 40 mA/cm^2^, the CE values for the LbL membranes assembled with *n* = 1 and *n* = 5 are 88.4% and 85.6%, respectively, surpassing those of the *n* = 0 membrane with 83.3%. The CE values for all membranes increased as the current density increased and such an increase can be attributed to the shorter time available for vanadium ions to permeate through the membranes at higher current densities, resulting in reduced cross-contamination and side reactions [58].

Previous research has shown that as current densities increase, there is a decrease in VE. Similarly, in this study, we observed a decrease in VE values as the current density increased. This decrease in VE can be attributed to increased ohmic loss, activation loss, and mass transfer loss. These losses occur due to the resistance encountered by the flow of ions. Additionally, the presence of microbubbles on the electrode surface, which are typically formed by side reactions such as hydrogen and oxygen evolution and water splitting, can also contribute to these resistances [59].

When comparing the LbL membranes used in the present study, we noticed that the VE value for the *n* = 1 membrane is higher than that of the *n* = 0 and *n* = 5 membranes, despite having the lowest proton conductivity. One possible explanation for this inconsistency is that the proton conductivity measurement by EIS did not involve any medium such as sulfuric acid or DI water. Instead, the membranes were only immersed in sulfuric acid for at least 48 h before the measurement, which may not fully replicate the actual vanadium electrolyte environment. Therefore, it is speculated that selectivity, which considers both proton conductivity and vanadium permeability, plays an equally important role in determining the overall efficiency of the membrane in a real VRFB system. Since the overall performance is accurately explained by CE and VE, which contribute to EE, it can be concluded that the *n* = 1 membrane consistently outperforms the others, exhibiting the highest selectivity and EE value.

The stability of the membranes chosen was assessed by subjecting them to continuous cycling at a current density of 40 mA/cm^2^ for 100 cycles, and the findings are displayed in Figure 7(b). The results demonstrate that the CE of the LbL membranes consistently outperforms the *n* = 0 membrane. The CE of the LbL membranes remained relatively stable at around 88% for the *n* = 1 membrane and approximately 85% for the *n* = 5 membrane, indicating favorable chemical stability of the deposited PEI/GO bilayers. Regarding VE, both the *n* = 0 and *n* = 5 membranes exhibited a slight decrease, while the *n* = 1 membrane maintained a stable value. Comparing the stability of the LbL membranes, it is evident that the *n* = 1 membrane is more stable than the *n* = 5 membrane. The slight decrease in VE for both *n* = 0 and *n* = 5 could be attributed to membrane fouling, suggesting a potential accumulation of vanadium ions near the membrane surface. This accumulation leads to a reduction in vanadium suppression over time and an increase in AR, resulting in a decline in VE [58,60]. However, it is expected that the application of the PEI/GO bilayers on the membrane surface will decrease the rate of accumulation, thereby mitigating these effects.

**Figure 8.**
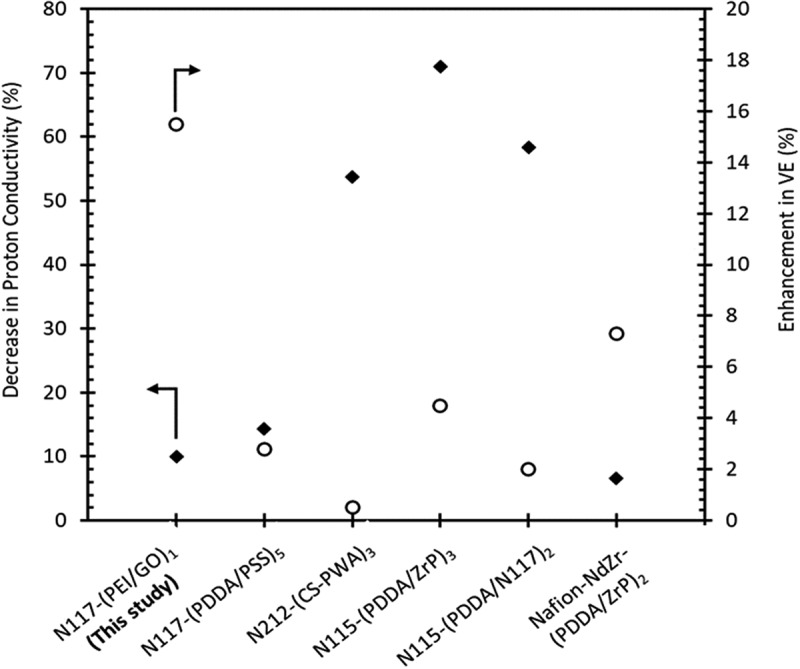
Comparison of decrease in proton conductivity and enhancement in VE for LbL membranes using different polyelectrolyte pairs.

In order to test the hypothesis of this study, which proposes that the use of PEI/GO bilayers will maintain a high level of proton conductivity and improve the feasibility of the membrane through the LbL approach, a comparative analysis was performed and the data are presented in Figure 8. The analysis focused on determining the percentage decrease in proton conductivity and the corresponding increase in VE for each optimized value of *n*, in comparison to Nafion-based LbL membranes modified with polyanion bilayers reported in previous studies [15,19–21,47]. The present membranes with PEI/GO bilayers exhibited the smallest reduction in proton conductivity while achieving the highest enhancement among the other LbL membranes. Notably, the N115-(PDDA/ZrP)_3_ membrane shows the greatest decrease in proton conductivity, followed by N115-(PDDA/117)_2_ and N212-(CS-PWA)_3_. Despite the significant decrease in proton conductivity, they only demonstrate minor enhancements in VE ranging from 0.5% to 6.0%.

## 4. Conclusions

A highly selective PFSA membrane was developed for VRFB application based on Nafion 117. The modification of N117 membrane was successfully achieved using PEI/GO bilayers through the LbL modification technique. The prepared membranes exhibited favourable physicochemical properties by combining acceptable ion IEC, WU, and SR. Among the LbL membranes, the *n* = 1 membrane demonstrated the highest selectivity (19.2 × 104 S.min/cm^3^), indicating a balanced combination of proton conductivity and vanadium permeability. During VRFB testing, the *n* = 1 membrane consistently outperformed the other multi-bilayer membranes, showing longer self-discharge time (approximately 2.5 times longer than *n* = 0 membrane) and better capacity. Additionally, the *n* = 1 membrane exhibited a 4% to 6% improvement in CE and a 7% to 15% improvement in VE. Remarkably, it maintains outstanding stability over 100 VRFB cycles. These findings suggest that the LbL self-assembly technique using PEI/GO holds promising potential for enhancing selectivity in VRFB applications.
